# Mechanisms of Anergic Inflammatory Response in Nasopharyngeal Carcinoma Cells Despite Ubiquitous Constitutive NF-κB Activation

**DOI:** 10.3389/fcell.2022.861916

**Published:** 2022-07-22

**Authors:** Xiaoying Zhou, Liudmila Matskova, Shixing Zheng, Xiaoxia Wang, Yifang Wang, Xue Xiao, Yingxi Mo, Marleen Wölke, Limei Li, Qian Zheng, Guangwu Huang, Zhe Zhang, Ingemar Ernberg

**Affiliations:** ^1^ Department of Microbiology, Tumor and Cell Biology, Karolinska Institutet, Solna, Sweden; ^2^ Life Science Institute, Guangxi Medical University, Nanning, China; ^3^ Key Laboratory of Early Prevention and Treatment for Regional High-Frequency Tumor (Guangxi Medical University), Ministry of Education, Nanning, China; ^4^ ENT Institute and Department of Otorhinolaryngology, Eye and ENT Hospital, Fudan University, Shanghai, China; ^5^ Department of Otolaryngology-Head and Neck Surgery, First Affiliated Hospital of Guangxi Medical University, Nanning, China; ^6^ Department of Research, Affiliated Tumor Hospital of Guangxi Medical University, Nanning, China

**Keywords:** nasopharyngeal carcinoma, microbes, NF-κB, lipid droplets, LSD1

## Abstract

Commensal microbes cross talk with their colonized mucosa. We show that microbes and their cell wall components induce an inflammatory response in cultured human mucosal cells derived from the nonmalignant nasopharyngeal epithelium (NNE) cells *in vitro*. NNE cells show significant induction of NF-κB with nuclear shuttling and inflammatory gene response when exposed to Gram-positive bacteria (streptococci) or peptidoglycan (PGN), a component of the Gram-positive bacterial cell wall. This response is abrogated in nasopharyngeal carcinoma (NPC)–derived cell lines. The inflammatory response induced by NF-κB signaling was blocked at two levels in the tumor-derived cells. We found that NF-κB was largely trapped in lipid droplets (LDs) in the cytoplasm of the NPC-derived cells, while the increased expression of lysine-specific histone demethylase 1 (LSD1, a repressive nuclear factor) reduces the response mediated by remaining NF-κB at the promoters responding to inflammatory stimuli. This refractory response in NPC cells might be a consequence of long-term exposure to microbes *in vivo* during carcinogenic progression. It may contribute to the decreased antitumor immune responses in NPC, among others despite heavy T-helper cell infiltration, and thus facilitate tumor progression.

## Introduction

Nasopharyngeal carcinoma (NPC) arises in the mucosal epithelium in the epipharynx. Viral (Epstein–Barr virus, EBV), genetic, and environmental factors provide the significantly increased risk of NPC not only in the endemic areas of particularly South East Asia but also North Africa and Greenland (Chen et al., 2019; Xu et al., 2019). The mucosa of the epipharynx is exposed to the local microbial flora throughout life. The inflammatory response against both commensal microbes and pathogens is tightly controlled in normal host–microbe interactions, which does not preclude imbalances, resulting in chronic inflammation.

The ability to induce an adequately strong inflammatory response to invading microorganisms, to their virulence factors, and/or to damaged cells is essential for successful resolution of infections, repair of cell damage, and protection against a chronic inflammatory response. NF-κB activation leading to its shuttling to the nucleus is the predominant pathway for induction of these inflammatory genes.

Chronic inflammation is a well-established risk factor for cancer (Balkwill and Mantovani, 2001; Greten and Grivennikov, 2019). Studies in mouse models provide a solid support for induction of cancer in inflammatory environments (Arthur et al., 2012; Bongers et al., 2014; Viennois et al., 2017). In man, for example, the Hepatitis B virus and *Helicobacter pylori* induce persistent inflammatory environments preceding cancer development (Murata, 2018; Tang et al., 2018). Meta-analyses of epidemiologic studies have demonstrated significant reduction of common cancers in human subjects after intake of nonsteroidal anti-inflammatory drugs (NSAID) or aspirin for periods of 10 years or longer (García Rodríguez and Huerta-Alvarez, 2001; Flossmann and Rothwell, 2007; Rothwell et al., 2012).

Inflammation in the nasopharynx as a possible factor contributing to NPC has not received much attention until now. We suggest that this might be an important and partly overlooked factor in NPC risk and progression. Imbalances of the commensal microflorogenic metabolites are observed (Guha et al., 2007; Abnet et al., 2008; Rogers, 2011; Dejea et al., 2013). Cell wall components and factors released from these bacteria can directly modify the gene expression patterns of host epithelial cells by epigenetic means in the oronasal cavity, which may induce chronic inflammatory conditions both *via* direct cell–microbe contacts and release of soluble factors and may also provide sources of carcinogens (Chan et al., 2003; Nakajima et al., 2009; Takahashi et al., 2011). Here, we used *in vitro* model systems with nonmalignant nasopharyngeal and NPC-derived cell lines to explore whether exposure to microbes or microbial components may affect the activation of NF-κB and the expression of proinflammatory genes.

Temporal and/or spatial dysregulation of inflammatory responses may lead to chronic inflammation. Inflammation is typically initiated as an innate immune response to specific bacterial products through receptor-dependent mechanisms, with NF-κB playing an important role (Hoffmann and Baltimore, 2006). Toll-like receptors (TLRs) are membrane-bound sensors that mediate the recognition of microbial molecules to promote immune responses (Medzhitov, 2007). NF-κB is typically activated by components of bacterial cell walls such as peptidoglycan (PGN) and lipopolysaccharide (LPS) and agonists of TLR4 and TLR2, respectively. This leads to activation of NF-κB–regulated genes including proinflammatory cytokines (Poltorak et al., 1998; Manicassamy and Pulendran, 2009). NPCs are almost exclusively EBV-positive in the endemic areas. EBV is an established risk factor for NPC strongly vindicated by the recent molecular epidemiologic studies (Xu et al., 2019). EBV infection has been shown also to affect the interaction of microbes with host epithelial cells (Ito et al., 1980; Charriere et al., 1991; Liu et al., 1998).

Regulation of NF-κB has been studied extensively in NPC and in EBV infection, primarily due to efficient activation of NF-κB by EBV latent membrane protein 1 (LMP1) (Huen et al., 1995; Zhang et al., 2013). LMP1 is expressed in early NPC *in situ* lesions, as well as in 35%–65% of NPC tumors (Fåhraeus et al., 1988; Young et al., 1988). Thus, the LMP1 regulation of NF-κB is of major interest to the inflammatory response both in the initiation of NPC and during its progression.

The nuclear shuttling of NF-κB is necessary for NF-κB to exert its activation but can be blocked by cytoplasmic lipid accumulation (Yan et al., 2008). Lipid droplet (LD) accumulation has been observed in several tumor types (Swinnen et al., 2006). We previously described one mechanism of abnormal LD accumulation in NPC cells. We found that epigenetic inactivation of UbcH8 in NPC cells negatively regulates a key enzyme in lipid catabolism and release from LDs, adipose triglyceride lipase (ATGL), due to the ISG15ylating activity of UbcH8 (Zhou et al., 2014). Recently, we reported that EBV-encoded latent membrane protein 2A (LMP2A) promotes the motility of NPC cells by suppressing the expression of ATGL, therefore blocking the degradation of LDs (Zheng et al., 2020).

Lysine-specific demethylase 1 (LSD1) demethylates the di/tri-methylated lysine 4 of histone 3 (H3K4), which is a marker generally associated with transcriptional activity. LSD1 and histone deacetylase 1 (HDAC1) have been demonstrated to repress the expression of genes related to tumor immunity and inflammation (Shi et al., 2005; Janzer et al., 2012; Sheng et al., 2018; Qin et al., 2019). Chromatin modification by LSD1 at proinflammatory gene promoters reduces the effect of NF-κB at such promoters. Imbalances in the expression and degradation of LSD1, which result in its overexpression, have been reported in tumors (Hayami et al., 2011; Dalvi et al., 2019).

The purpose of this work is to examine the role of the nasopharyngeal microflora as an additional component in the pathogenesis of NPC by establishing and utilizing an *in vitro* cell line model. In this model, we investigated the possibility of whether bacterial cell wall components can induce an inflammatory response. We focused on the activation of inflammation by NF-κB signaling in NPC-derived cells. The observed anergy of NF-κB signaling in NPC tumors was further elucidated by linking it to lipid metabolism in NPC cells and to epigenetic regulators.

## Results

### Peptidoglycan Induces a Strong Inflammatory Response in Nonmalignant Nasopharyngeal Epithelium–Derived Cells

**FIGURE 1:**
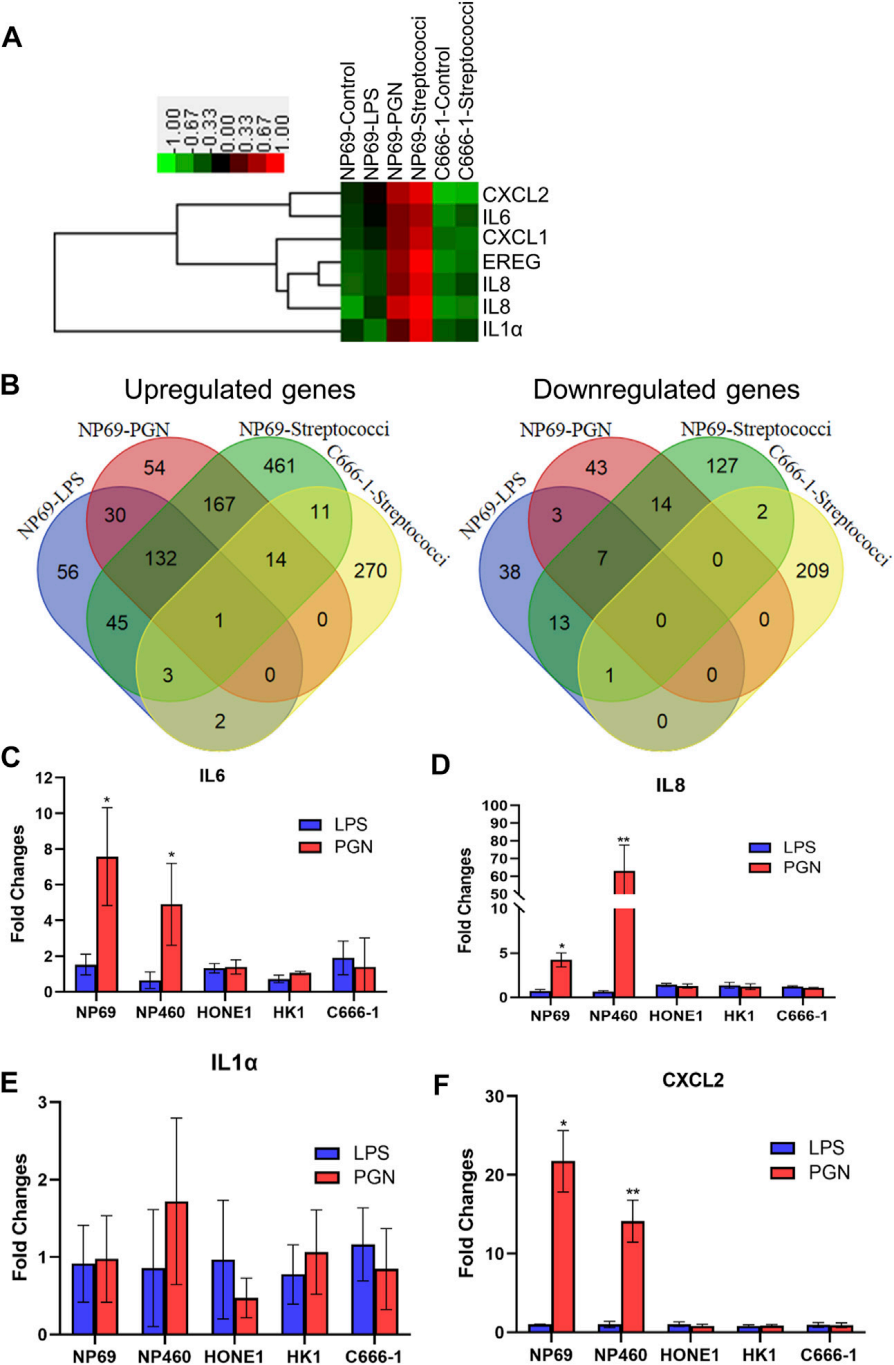
Differential inflammatory response of nonmalignant nasopharyngeal epithelial cells (NNEs) and malignant, NPC-derived cells upon exposure to peptidoglycan (PGN) or streptococci. **(A)** cDNA microarray analysis was carried out in NP69 cells, following treatment by lipopolysaccharide (LPS), PGN, or live streptococci. A heat map was generated for six genes represented by seven probe sets. **(B)** Venn diagram demonstrates the differentially expressed genes. The numbers represent the overlapping genes. **(C–F)** Real-time RT-PCR analysis confirming differential expression of proinflammatory cytokines IL1α, IL6, IL8, and CXCL2 in NNE (NP69 and NP460) and NPC cells (HONE1, HK1, and C666-1), exposed to LPS and PGN. A bar graph shows the fold changes in gene expression (mean ± s.d. expression in the treatment group over untreated). Each experiment was conducted in triplicate. *p < 0.05; **p < 0.01.

In order to map the response of NNE-derived cells to bacteria, we performed a systemic analysis of gene expression. NNE cells NP69 were exposed to LPS and PGN for 2 hours and the effects were then analyzed using cDNA microarray. We detected a strong response upon PGN treatment. By hierarchical clustering analysis, we found that most of the genes that were significantly upregulated after the treatment of NP69 cells with PGN were proinflammatory cytokines, such as IL1α, IL6, IL8, and CXCL2. In contrast, these genes were not affected in similarly treated C666-1 cells (Figure 1A).

Treatment of NNE cells with either PGN or streptococci (used as model Gram-positive bacteria) showed partly similar effects, with around 30% of the upregulated genes overlapping (Figure 1B). These genes were mainly known to respond to the TLR and Jak-Stat or participate in the cytokine–cytokine receptor signaling pathways, as suggested by KEGG pathway analysis (Supplementary Table S2). In contrast, there was no cross talk between genes activated by LPS and PGN, suggesting that the effect of Gram-negative and -positive bacterial cell wall components differs distinctly (Supplementary Table S3).

### The Inflammatory Response to Live Streptococci Was Curbed in Nasopharyngeal Carcinoma Cells

Interestingly, the responses to streptococci in the NPC-derived cell line C666-1 showed significantly fewer upregulated genes than that in NP69. A total of 834 genes were induced in NP69, while only 301 genes were induced in C666-1 (Figure 1B). Among these 301 genes, we found no genes involved in an inflammatory response, by KEGG pathway analysis. These genes were rather associated with the leukocyte transendothelial migration pathway, the phosphatidylinositol signaling system, and the Wnt signaling pathway. Only the tight junction pathway overlapped between C666-1 and NP69 after exposure to the bacteria (Supplementary Table S4).

### The Peptidoglycan Induces the Upregulation of Cytokines and Chemokines in Nonmalignant Nasopharyngeal Epithelium Cells but Not in Nasopharyngeal Carcinoma Cells

We further validated the mRNA expression of IL1α, IL6, IL8, and CXCL2 using real-time qPCR. The data confirmed that PGN, but not LPS, significantly stimulated the expression of proinflammatory cytokines and chemokines in NNE cells. In the NPC-derived cell lines (HONE1, HK1, and C666-1), the inflammatory response was much weaker, whether they were treated with PGN or LPS (Figures 1C–F).

### TLR2 and TLR4 Are Expressed at Similar Levels in Nonmalignant Nasopharyngeal Epithelium Cells and Nasopharyngeal Carcinoma Cells

**FIGURE 2:**
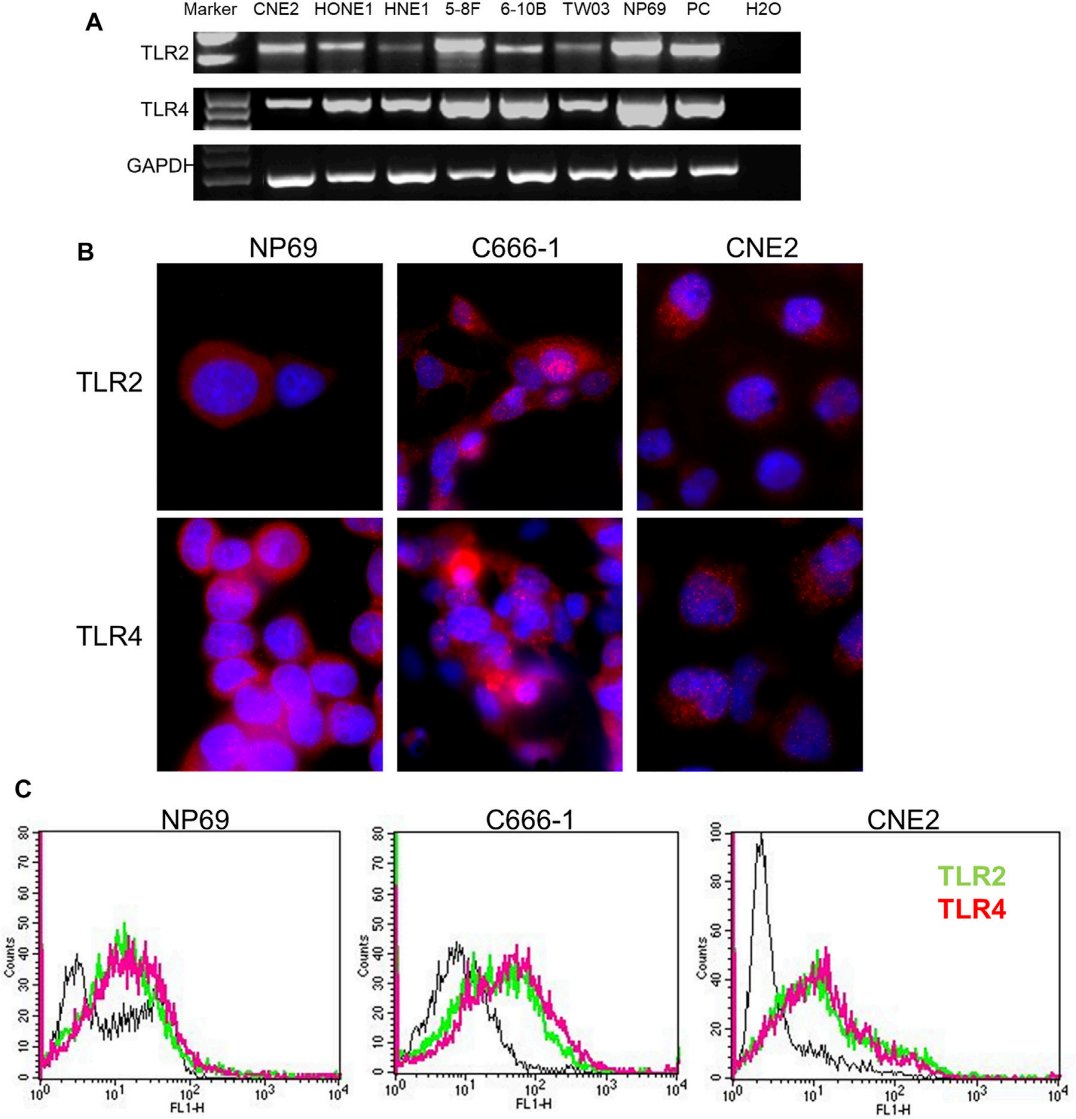
Expression of TLR2 and TLR4 in NNE and NPC cells. **(A)** RT-PCR analysis of TLR2 and TLR4 mRNA expression. **(B)** Immunofluorescence analysis of TLR2 and TLR4 protein expression. **(C)** Flow cytometry analysis of cell surface localization of TLR2 and TLR4.

TLR2 and TLR4 cell surface receptors are essential for sensing Gram-positive and -negative bacteria, respectively, in mammals. Therefore, we had to exclude that the differences in response were related to receptor expression. TLR2 and TLR4 were equally expressed in both NPC and NNE cells, both at the mRNA- and protein levels (Figures 2A,B). Flow cytometry analysis (FCM) of live cells also showed that both these TLRs were expressed at the cell surface (Figure 2C). Thus, the differential inflammatory response in NNE- and NPC-derived cells could not be explained by differences in TLR2- and TLR4-expression or localization.

### The Peptidoglycan Induces the Activation of NF-κB (p65) in Nonmalignant Nasopharyngeal Epithelium Cells but Not in Nasopharyngeal Carcinoma Cells

**FIGURE 3:**
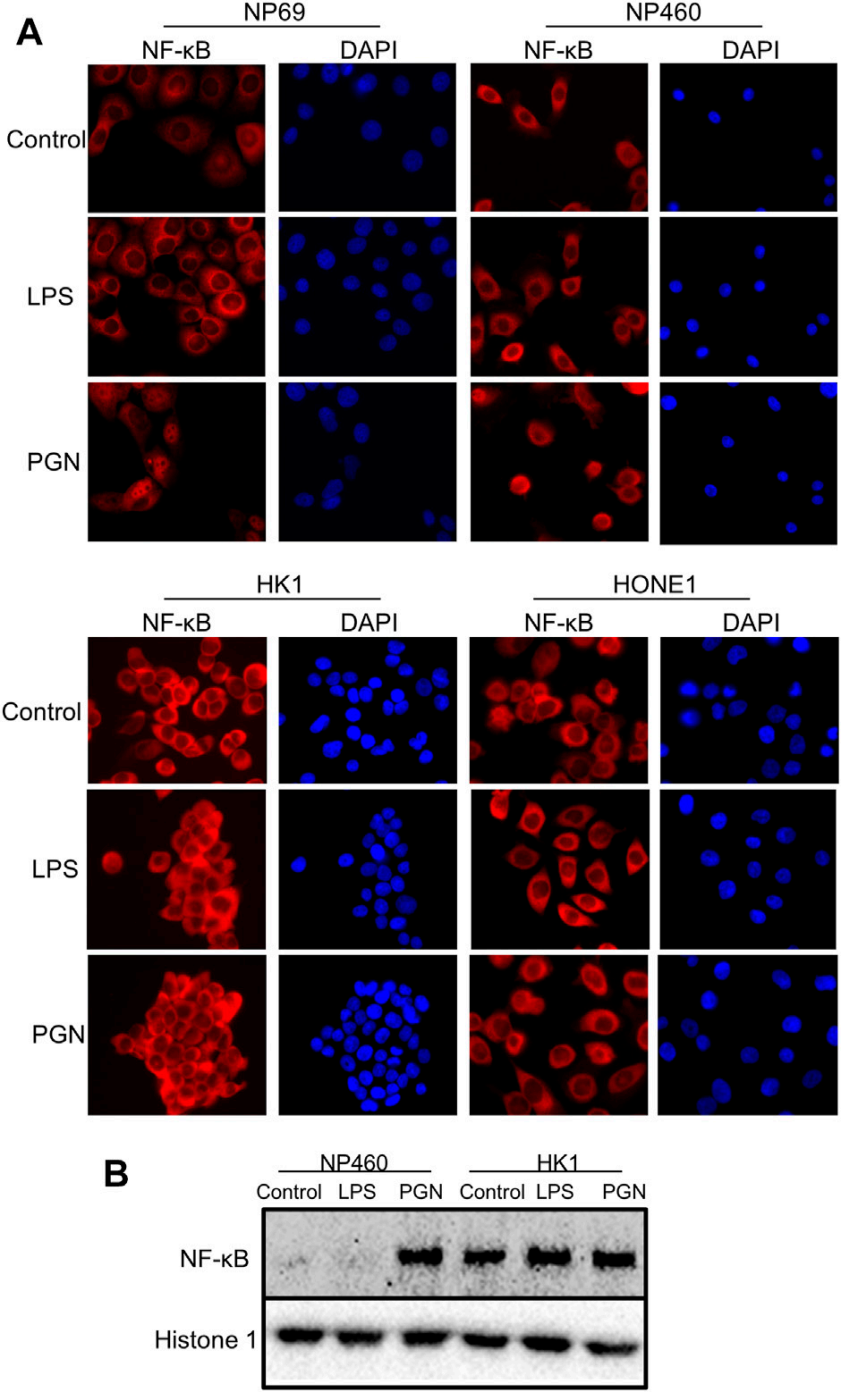
Differential NF-κB activation in normal and tumor cells. **(A)** Immunofluorescence analysis of activated, nuclear NF-κB (p65) fraction in NNE cell lines NP69 and NP460, versus NPC cell lines HONE1 and HK1, upon exposure to either 10 μg/ml LPS or 10 μg/ml PGN for 2 hours. Cell nuclei were visualized by DAPI. **(B)** Western blot analysis of nuclear NF-κB fractions in the NP460 and HK1, treated with LPS and PGN as previously mentioned.

NNE-derived cells were treated with LPS or PGN. Only PGN induced significant nuclear translocation of NF-κB (p65) as demonstrated by immunofluorescent staining (Figure 3A), indicating that the NF-κB signaling pathway can be activated by Gram-positive bacteria. In contrast, in NPC cells, we found NF-κB to be constitutively present in the nuclei and neither exposure to LPS nor to PGN caused any further nuclear translocation of NF-κB. Figure 3B shows NF-κB expression in nuclear fractions of non-malignant NP460 and NPC-derived HK1 cells. More NF-κB was found in the nuclear fraction of HK1 tumor cells than in NP460. Thus, PGN but not LPS treatment stimulated NF-κB nuclear shuttling in NP460 cells, while neither PGN nor LPS caused nuclear translocation of NF-κB in HK1 cells. Consistent with our microarray data, we found an induction of NF-κB activity in NNE-derived cells induced by PGN and live streptococci, but not in NPC cells. Cytoplasmic retention of NF-κB may provide one clue to explain the inability to further enhance the constitutive shuttling of NF-κB in NPC cells.

### Contribution of Lipid Droplet Accumulation in Nasopharyngeal Carcinoma Cells to NF-κB Localization

**FIGURE 4:**
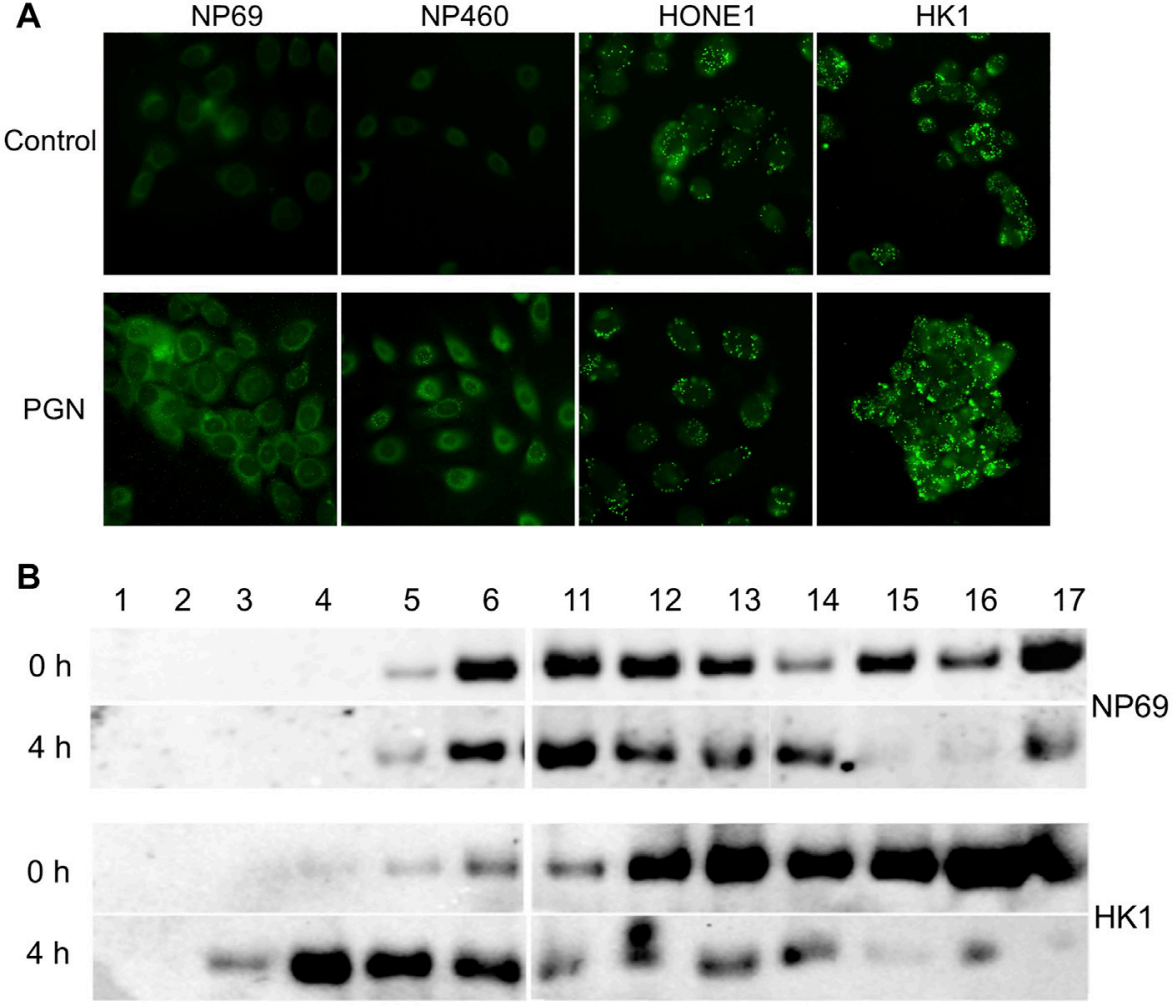
Lipid droplet (LD) accumulate in NPC cells. NF-κB is associated with LDs in NPC cells. **(A)** Immunofluorescent analysis of lipid content in NPE cell lines (NP69 and NP460) and NPC cell lines (HONE1 and HK1) USING a lipid-specific fluorescent dye BODIPY 493/503. **(B)** Cells were treated with 10µg/mL of PGN for 2 hours, the media was changed, and the cells were harvested at 0 and 4 h time points. The HK1 versus NP69 cells were fractionated on a discontinuous density gradient to separate lipid-containing fractions. Fractions were analyzed using Western blot for the expression of NF-κB (p65).

Previously, we have reported excessive LD accumulation in NPC cells (Zhou et al., 2014). Here, we observe that PGN induces lipid accumulation in nonmalignant cells, while in NPC cells, LDs are maintained at a steady-state level (Figure 4A). To investigate whether lipid accumulation is associated with cytoplasmic localization of NF-κB, we compared the NF-κB distribution in lipid fractions from HK1 and NP69 cells by Western blot analysis of OptiPrep density gradient fractions, after ultracentrifugation of protein lysates (Figure 4B). The cells were treated with PGN for 2 hours, the medium was changed, and the cells were harvested at several later time points. NF-κB in HK1 cells was detected floating to higher fractions of the gradient than NF-κB from NP69 cells already after 4 hours of cell exposure to bacterial factor PGN. After 8 hours and overnight, the distribution of NF-κB in NP69 cells in the density gradient had completely returned to the zero time point profile after exposure of cells to PGN, while NF-κB from HK1 cells dissociated slower from lipids (Supplementary Figure S1) and was found at the bottom of the gradient. Thus, lipid accumulation correlated to the entrapment of NF-κB in the cytoplasmic lipid fractions.

**FIGURE 5:**
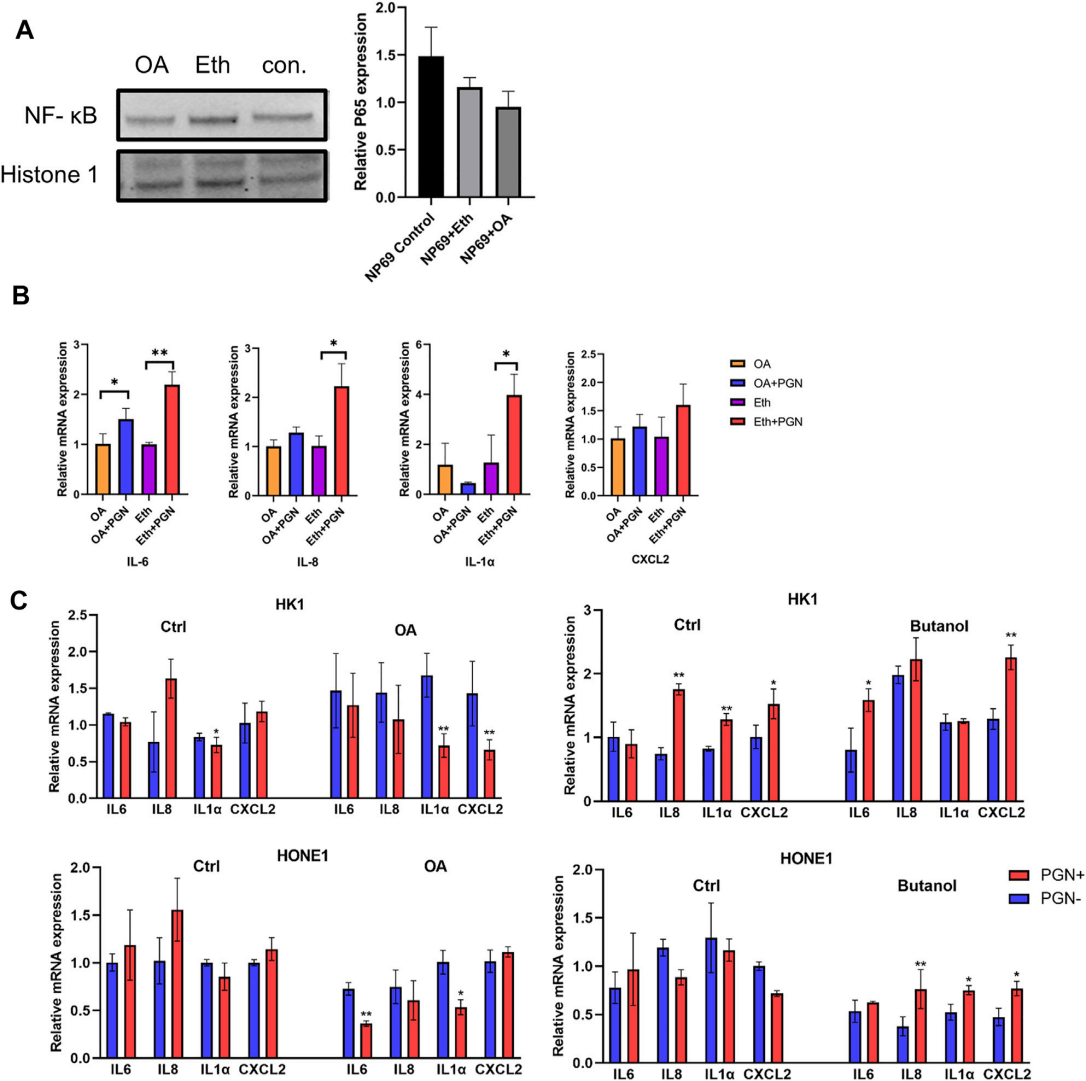
Intracellular amount of LDs affects the nuclear translocation of NF-κB (p65). **(A)** NNE cell line NP69 was treated with oleic acid (OA) for 48 h to increase intracellular LDs. The NF-κB (p65) expression in the nuclear fraction from NP69 cells was analyzed using Western blot. **(B–C)**: Transcriptional level of pro-inflammatory cytokines IL6, IL8, IL1α, and CXCL2 was negatively correlated with the intracellular amount of LDs in noncancerous nasopharyngeal epithelial cells and NPC-derived cell lines, before and after stimulation with 10µg/mL of PGN for 2 h *p < 0.05; **p < 0.01.

To further confirm the role of LDs in NF-κB translocation, we perturbed lipid formation in NP69 cells and evaluated the expression of inflammatory genes using qPCR analysis. We have previously shown that treatment of NPC cells with oleic acid (OA) increases the cytoplasmic LDs, while treatment with butanol leads to a decrease in LDs (Zheng et al., 2020). We investigated NF-κB (p65) localization in noncancerous nasopharyngeal epithelial cell lines after treatment with OA. The nuclear fraction of NF-κB (p65) in NP69 cells treated with OA was decreased when compared to the control, although this was not statistically significant (Figure 5A). Remarkably, the induction of NF-κB (p65) downstream proinflammatory genes was suppressed when increasing the LD accumulation by OA and stimulated by PGN (Figure 5B). Moreover, the induction of inflammatory genes after stimulation with PGN was stronger if NPC-derived cells were treated with butanol but decreased after treatment with OA (Figure 5C). The amount of the nuclear fraction of NF-κB (p65) and the cellular inflammatory response showed a negative correlation with the level of LDs.

### Contribution of Lysine-Specific Histone Demethylase 1 to Repression of the Inflammatory Response

**FIGURE 6:**
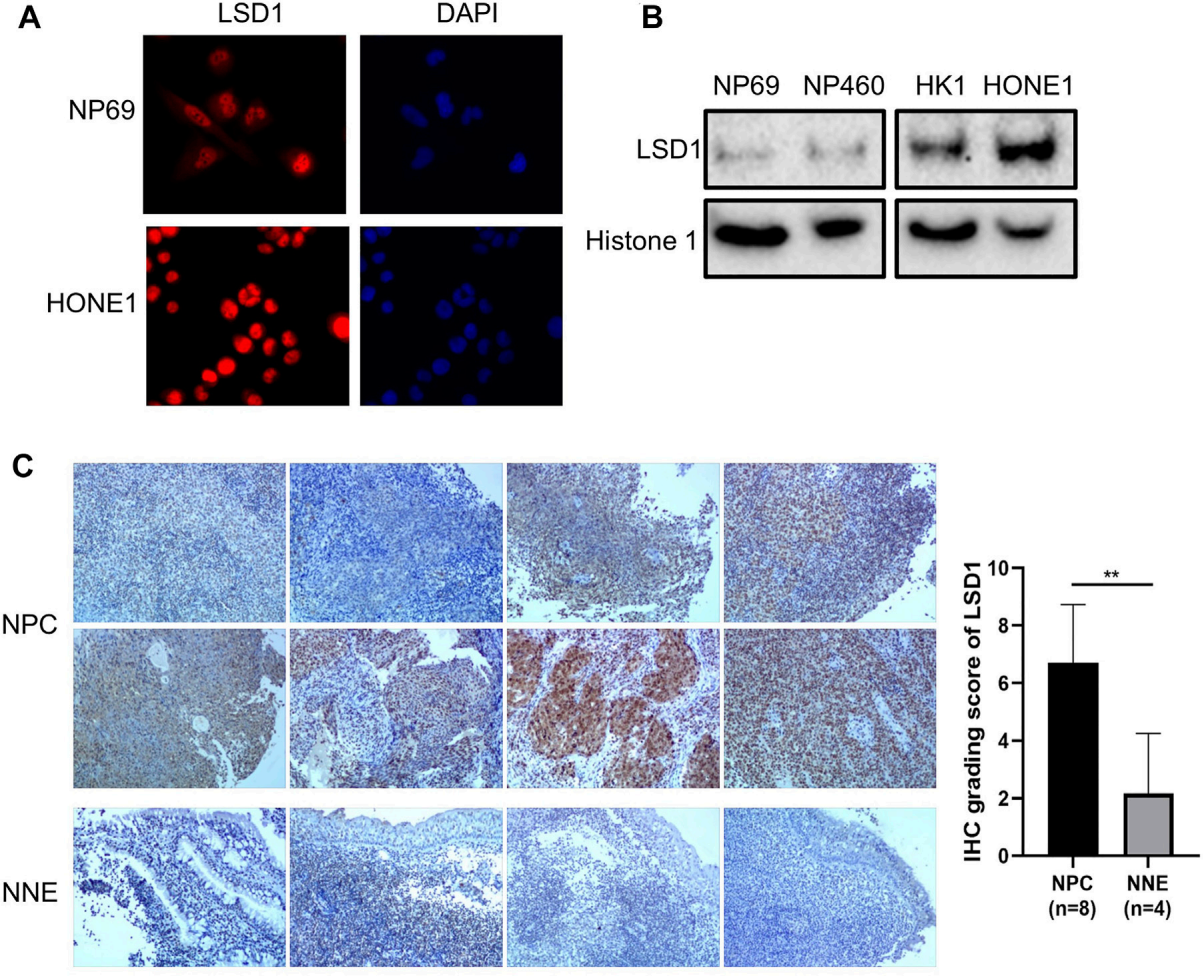
Abnormally increased LSD1 expression in NPC cells. **(A)** Immunofluorescence analysis of LSD1 (red) in NP69 and HONE1 cells. Cellular nuclei were counter-stained with DAPI. **(B)** Western blot analysis for LSD1 expression in cellular fractions of NNE versus NPC cell lines. Histone 1 was used as an endogenous control of protein loading for the nuclear fractions. **(C)** Immunohistochemistry staining of LSD1 in NPC (*n* = 8) and NNE tissues (*n* = 4).

**FIGURE 7:**
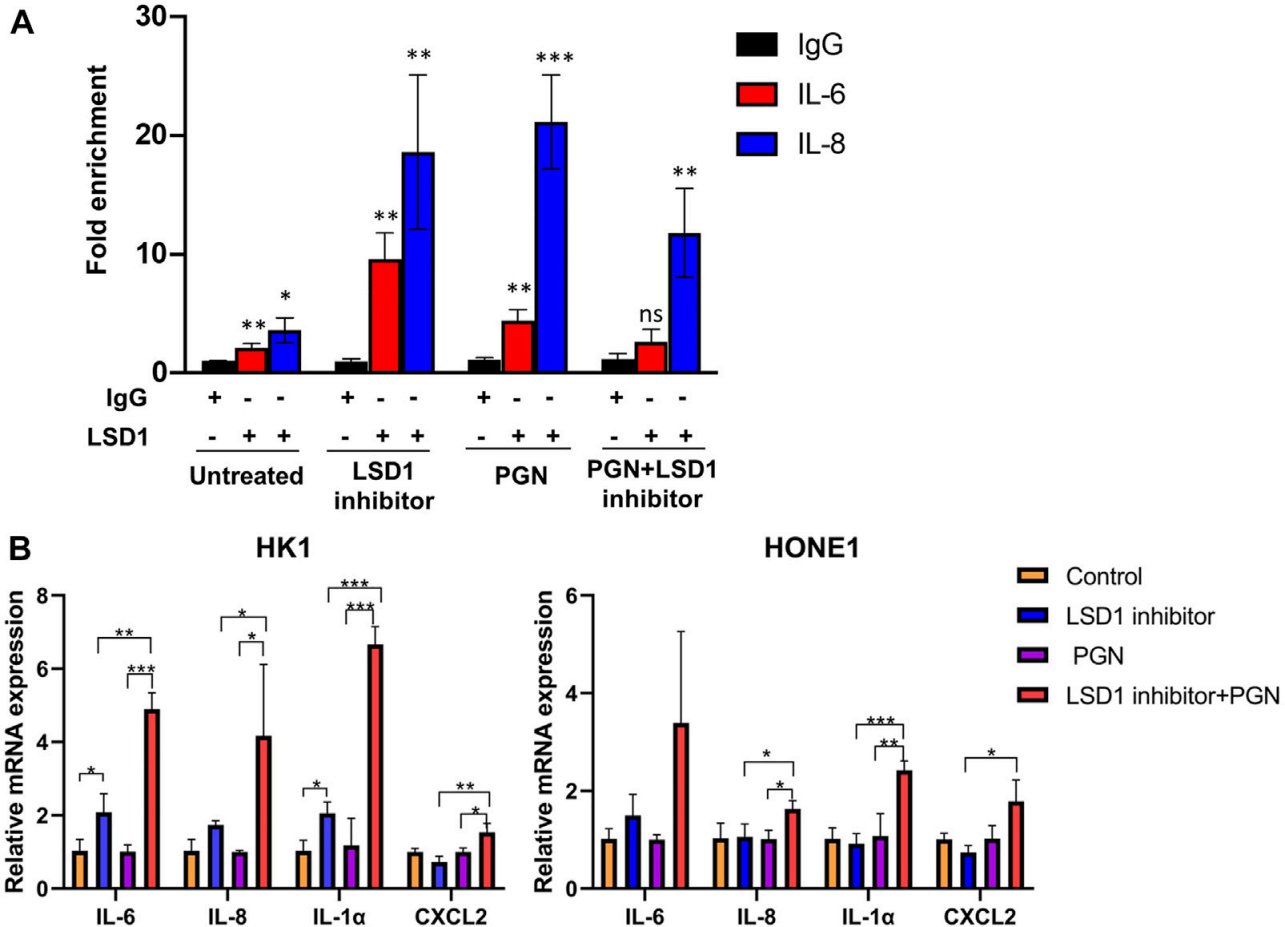
LSD1 inhibits the transcription of pro-inflammatory genes in NPC-derived cell lines. **(A)** LSD1 ChIP analysis of IL6 and IL8 promoter occupancy in C666-1 cell. The bar graph represents fold enrichment in the LSD1 antibody binding to the IL6 and IL8 promoters after the treatment of NPC cell line C666-1 with LSD1 inhibitor at 6 nM, PGN at 1 µg/ml, or together overnight, in relation to IgG binding. **(B)** Real-time RT-qPCR analysis of proinflammatory gene expression in HK1 and HONE1 cells before and after the treatment with LSD1 inhibitor overnight and followed by stimulation with PGN for two hours *p < 0.05; **p < 0.01; ***p < 0.001.

To evaluate the activity of nuclear NF-κB in NPC cells, we investigated the role of the repressive nuclear factor LSD1 in NNE- and NPC-derived cells. The histone modifier LSD1 is highly expressed in the nuclei of tumor cells, in contrast to NNE cells (Figures 6A,B). The aberrant expression of LSD1 was confirmed at the tissue level as well. In NPC tissues (*n* = 8), we observed a higher expression of LSD1 with a nuclear localization than the NNE (*n* = 4) (Figure 6C). In the tissue array, including 131 NPC tissue samples, we found that the positive staining ratio of LSD1 was 86% (113/131) (Supplementary Figure S2A). In 36 cases, we could compare NPC with adjacent matched normal epithelium, and in these, the protein expression level of LSD1 was remarkably higher in NPC than in the matched normal tissue (Supplementary Figure S2B). In addition, we performed chromatin immunoprecipitation (ChIP) assays with LSD1 antibody to assess whether LSD1 could bind directly to the promoter of proinflammatory genes and thus affect their accessibility for activating transcriptional complexes. We found that LSD1 binds directly to the IL6 and IL8 gene promoters in C666-1 cells (Figure 7A). The bacterial factor PGN increased LSD1 binding to the IL6 and IL8 cytokine promoters. Inhibition of the catalytic activity of LSD1 also resulted in increased LSD1 binding. Co-treatment of C666-1 cells with LSD1 inhibitor together with PGN showed no synergistic effect. Furthermore, we found that the chemical inhibition of the LSD1 catalytic activity upregulated the transcription of proinflammatory genes (IL1α, IL6, IL8, and CXCL2) which were significantly further enhanced after stimulation with PGN (Figure 7B). Our data suggest that the increased expression of LSD1 might contribute to the inhibition of the inflammatory response in NPC cells.

## Discussion

The interplay between the commensal microflora and its host affects the local tissue biology, with long-term consequences for disease risk and progression (Tlaskalová-Hogenová et al., 2011). NPC is a cancer that arises in the human nasopharynx, an ecological niche that is colonized with a diverse community of commensal bacteria, the nasopharyngeal microbiota, the composition of which is affected by environmental factors including diet and smoking (Ling et al., 2013). The local epithelium senses and responds to the microbes and their metabolites. In all normal microflora, there is a challenging balance between the induction of a local inflammatory response on the one hand and tolerance to the microflora on the other. A persistent imbalance between the local microflora and the host mucosa might result in chronic inflammation (Shacter and Weitzman, 2002). Thus, it is important to address the role of the nasopharyngeal microflora as an additional component in the pathogenesis of NPC.

To elucidate this, we exploited *in vitro* cell line models, in which the effects of whole microbes and the microbial cell wall components LPS and PGN can be studied on nonmalignant and malignant cells. Exposure of nonmalignant human nasopharyngeal mucosal cells to microbes or PGN *in vitro* resulted in a strong inflammatory response as shown by NF-κB activation and induction of cytokine and chemokine production. On the contrary, NPC-derived cells were unable to mount an inflammatory response to either of the microbial subcomponents LPS or PGN. By whole-genome expression profiling, we found that the proinflammatory genes accounted for the largest differences between the two cell types.

The inflammatory response induced by NF-κB signaling was blocked at two levels in the tumor-derived cells. We found that NF-κB was largely trapped in lipid droplets (LDs) in the cytoplasm of the NPC-derived cells, while the increased expression of lysine-specific histone demethylase 1 (LSD1, a repressive nuclear factor) reduces the response mediated by remaining NF-κB at the promoters responding to inflammatory stimuli.

A link between LD accumulation and NF-κB activity has been established in T lymphocytes (Yan et al., 2008). In these cells, lipid accumulation was only transient. The accumulation of LDs is frequently observed not only in leukocytes during inflammatory responses but also in epithelial cells (Bozza and Viola, 2010). For cancer cells, on the contrary, it is common to have continuous high levels of lipids (Cheng et al., 2018; Ray and Roy, 2018; Zadra et al., 2019). We have demonstrated that LDs accumulate in NPC cells (Zhou et al., 2014). LDs represent deposits of neutral lipids, surrounded by proteins, which participate in lipid metabolism (Brasaemle et al., 1997), membrane trafficking (Liu et al., 2004), and intracellular signaling (Yu et al., 2000). We found that PGN promoted LD formation in NNE cells, while no such effect was observed after LPS treatment, which correlates with the difference in the inflammatory responses between PGN and LPS that we observed in our epithelial cell lines. We show here that NF-κB colocalizes predominantly with the lipid fraction in tumor cells in contrast to nonmalignant cells.

NPC tumors show classical signs of NF-κB activation, such as I-κB-phosphorylation, cleavage of the p105 precursor, and nuclear translocation (You et al., 2019). We also see increased levels of NF-κB in the tumor cells, in spite of that much of NF-κB is trapped in the cytoplasmic/LD fraction. In order to address the question of why a lot of NF-κB still is localized to the nuclei of NPC-derived cells, in spite of a lot of cytoplasmic trapping, but does not activate the transcription of proinflammatory cytokines, we explored transcriptional cofactors controlling NF-κB activation.

LSD1 is an H3K4me demethylase, often upregulated in tumors (Wang et al., 2011). It is also responsible for stabilizing DNMT1 (Wang et al., 2009), which is essential for cancer progenitor cell maintenance and tumorigenesis (Pathania et al., 2015). It blocks the activation of proinflammatory cytokines like IL1, IL6, and IL8 (Janzer et al., 2012). Promoters of proinflammatory genes in NPC-derived cells showed enhanced LSD1-binding in line with that these genes were downregulated. LSD1 was exclusively localized to the nuclei and more abundant in NPC cells than nonmalignant cells. Increased binding of LSD1 to proinflammatory gene promoters (IL6 and IL8) after exposure to the bacterial factor PGN would support that this negative transcriptional modifier LSD1 blocks any induction of an inflammatory response in NPC-derived cells. It is tempting to speculate that in response to an inflammatory response, the tumor cells assemble both positive (NF-κB) and negative (LSD1) transcription factors on the promoters of proinflammatory genes, followed by demethylation of H3K4me3 by LSD1, leading to blockade of NF-κB binding. Thus, the level of transcription of IL6 and IL8 messages depends on the availability of the nuclear NF-κB and the catalytic activity of LSD1. Using a ChIP assay, we showed that LSD1 was indeed localized at the promoter of proinflammatory genes, and by a catalytic inhibitor, we could also show that LSD 1 controlled their expression.

Normal mucosal cells have a relatively short turn-over time *in vivo*, that is, a single cell will not be exposed to microbes for more than approximately maximum 1–2 weeks until they are terminally differentiated and exfoliated. In contrast, tumor cell clones *in vivo*, being immortalized, live for a long time and with constant exposure to the local microflora over this extended time. This might explain why tumor cells develop resistance to the constant proinflammatory environment from the microbiota.

Although we demonstrated blocks of NF-κB–driven inflammatory pathways in NPC cells, this does not preclude a role of chronic inflammation at the onset of NPC before the cancer cells have adapted to neglect the inflammatory pressure.

NPC tissues are massively invaded by T-lymphocytes, particularly of CD4-type, and their interplay with the cancer cells has been suggested to play a role in pathogenesis (Ferradini et al., 1991). Recent data also suggest that the local environment at the onset of NPC might be affected by consistent changes in the microflora together with the effects of EBV on inflammatory pathways (Debelius et al., 2020; Jin et al., 2020). An early inflammation might have changed to the anergic situation during the progression to cancer.

In conclusion, NF-κB signaling in NPC-derived tumor cells is anergic for two reasons. NF-κB is trapped in cytoplasmic LDs, while the fraction that overcomes this and translocates to the nucleus is blocked by the overexpression of the histone modifier LSD1 in tumor cells. On the contrary, in normal cells, NF-κB is active in response to PGN even if NF-κB transiently binds to the increased cytoplasmic lipids.

At least two different molecular mechanisms contribute to the impaired inflammatory response to microbial subcomponents in NPC-derived tumor cells. Our findings are relevant to the further elucidation of the role that host–microbe interactions may play in NPC pathogenesis and risk assessment.

## Materials and Methods

### Ethical Statement

All procedures followed the ethical standards of the Helsinki Declaration (1964, amended 2008) of the World Medical Association. All participants were informed about the aim, methodology, and possible risks of the study; informed consent was signed by each donor. The design of this study was approved by the Research Ethics Committee of Guangxi Medical University (Nanning, China).

### Human Tissues

Primary NPC tumor tissues were obtained from eight diagnosed and untreated patient cases (informed consent was signed by each donor) in the Department of Otolaryngology-Head and Neck Surgery, First Affiliated Hospital of Guangxi Medical University (Nanning, China). Four normal nasopharyngeal epithelial tissues were used as control.

A tissue microarray including 131 NPC tissue samples was purchased from Shanghai Outdo Biotech Co., Ltd. (Shanghai, China; Cat No: HNasN132Su01).

### Cell Lines and Bacterial Strains

*In vitro* transformed, NNE-derived cell lines NP69-SV40 and NP460 hTERT were maintained in a defined keratinocyte serum-free medium at 37°C in an atmosphere of 5% CO_2_ (Li et al., 2006). NPC-derived cell lines C666-1, HK1, HONE1, CNE2, HNE1, 5–8F, 6–10B, and TW03 were routinely cultivated in IMDM medium (Invitrogen, Carlsbad, CA., United States) containing 10% fetal calf serum (HyClone, United Kingdom Ltd., Northumberland, United Kingdom). Alpha-hemolytic streptococci were cultured on blood agar plates and further expanded by culturing in brain heart infusion broth (BHI, Sigma, St. Louis, Mo, United States). Before experimental use, streptococci were inoculated in 5 ml of BHI and incubated at 37°C until they reached the mid-logarithmic phase (optical density of 1.0 at 600 nm). Subsequently, streptococci were centrifuged and resuspended in pre-warmed PBS to the desired cell density for inoculation on cell lines at a multiplicity of infection (MOI) of 100.

### Reagents, Plasmid, and Antibodies

Lipopolysaccharides (LPS, L2630) and peptidoglycan (PGN, 69554) were purchased from Sigma (St. Louis, Mo, United States). TNF-α recombinant human protein (PHC3015) was obtained from Life Technologies (Rockville, MD, United States). LSD1 Inhibitor IV, RN-1, HCl (489479) was obtained from Millipore (Billerica, MA, United States). Antibodies and fluorescent dyes used in this study were anti-TLR2 (H-175), anti-TLR4 (M-300), anti-NF-κB (p65), anti-GAPDH (6C5) from Santa Cruz (CA, United States), anti-LSD1 from Abcam (Cambridge, MA, United Kingdom), and anti-ganglioside GM1 (LS-C66414) from LifeSpan Biosciences (Seattle, WA, United States). Secondary antibodies used were anti-rabbit/mouse IgG-HRP conjugate (Bio-Rad Laboratories, Hercules, CA, United States) and Alexa fluor 594 goat anti-rabbit IgG (H + L) (A-11037, Life Technology). Protein A/G plus-agarose (sc-2003) was obtained from Santa Cruz. DAPI (D1306) was obtained from Life Technology. Lipid droplet dye BODIPY (D3922, Molecular Probes, Carlsbad, California, United States) was diluted to a final/working concentration of 1 μg/ml and applied on slides or tissue sections for 30 min at room temperature.

### cDNA Microarray Analysis

The array platform used was Affymetrix Cartridge HG-U133 plus 2.0 (Santa Clara, CA, United States); one microarray was used for each cell line and each treatment. Array hybridization and basic data processing were performed at the Bioinformatics and Expression Analysis Core facility at Karolinska Institute, Stockholm, Sweden. The raw data processing was performed in the Expression Console from Affymetrix. The heat map was generated with Cluster and TreeView software. The KEGG signaling pathway was analyzed using the HyperSet website tool: network analysis made practical (https://research.scilifelab.se/andrej_alexeyenko/HyperSet/).

### Quantitative Real-Time PCR

RNA was purified using an RNeasy mini kit (QIAGEN, Valencia, CA, United States), and cDNA was prepared using the RevertAid First-Strand cDNA Synthesis Kit (Fermentas, Ontario, Canada). Quantitative real-time PCR (qPCR) was performed using StepONE plus system with a two-step PCR amplification using SYBR Green (Invitrogen, Carlsbad, CA, United States). Expression levels were normalized to the housekeeping gene GAPDH. The primers used for SYBR Green reactions are listed in Supplementary Table S1.

### Flow Cytometry Analysis

To analyze the surface expression of TLR2 and TLR4 in nasopharyngeal epithelial cell lines, adhesive cells were trypsinized, washed with PBS twice, and resuspended in 100 µl PBS. Primary antibody was added at 1:20 dilution and incubated for 1 hour at 4°C, followed by secondary fluorescent antibody diluted similarly, for another one hour at room temperature in the dark. Fluorescence was measured and analyzed on a flow cytometer with CellQuest software (BD FACS Calibur, San Jose, CA, United States).

### Western Blot Analysis and Immunofluorescence Staining

Nuclear proteins were isolated using a ProteoExtract^®^ Subcellular Proteome Extraction Kit (539790, Millipore, State, Country). The nuclear protein fraction was separated using SDS-PAGE and analyzed using a conventional Western blot assay as described elsewhere (Zhou et al., 2014). Immunofluorescence staining was performed as follows. A total number of 50,000 cells were seeded in six wells and allowed to attach overnight. Adherent cells were fixed with 4% formaldehyde for 15 min, permeabilized with 0.5% Triton X-100 for ten minutes, and blocked with 5% BSA for 30 min. They were incubated with primary antibodies at 4°C overnight. Subsequently, Alexa Fluor 568 labeled antibody was applied for 1 h at room temperature. Cell nuclei were counterstained with DAPI. Immunofluorescence images were acquired using a Leica DMRE microscope with HiPic software (Leica, Bensheim, Germany).

### Immunohistochemistry Staining

LSD1 protein expression was detected using immunohistochemical (IHC) staining. The tissue antigen retrieval was carried out by heating the tissue sections at 100°C in sodium citrate solution (pH 6.0) for three minutes. The tissue microarray was incubated with primary antibodies against LSD1 at 4°C overnight. Next, the Sp-9000 detection Kit (ZSDB-BIO, Beijing, China) was used for subsequent steps according to the instructions. Diaminobenzene (DAB) reagent (ZLI-9018, ZSGB-BIO, Beijing, China) was used as the chromogen, and hematoxylin was used for the nuclear counterstain. Finally, the slices were dehydrated, cleaned, and sealed. Images were acquired using a microscope (C-5050, Olympus, Japan).

The staining results were independently evaluated by two pathologists without the knowledge of sample source and clinical information. The number of cells in the 40 × 10 field of vision of five microscopes was randomly selected to evaluate the percentage of positive cells and staining intensity. The percentage of positive cells and staining intensity were multiplied to obtain the final score for each tissue section. The score of 0–1 is negative, 1–5 was considered weakly positive, 5–8 was considered positive, and more than 8 is considered strong positive. Take the protein expression of the positive and strong positive cancer nests and adjacent tissues as control (14 cases had only cancer nests, no adjacent tissues.)

GraphPad Prism 6 software was used for the overall survival (OS) curve. Statistical analysis was performed using the Statistical Program for Social Sciences (SPSS) 22.0 software.

### Lipid Fractionation

Cell lysis was performed in 1 ml of TXNE (50 mM Tris-HCl [pH 7.4], 150 mM NaCl, 5 mM EDTA, and 0.1% Triton X-100) with 10 μg of pepstatin A (Sigma) per mL and protease inhibitors (Complete; Roche Diagnostic Systems, Mannheim, Germany) at 4°C for a ten-cm diameter plate of HK1 cells, essentially as described previously (Matskova et al., 2001). Optiprep (Nycomed A/S, Oslo, Norway) gradients were made in three steps: the bottom step, containing the cell lysate, was 35% Optiprep (1.5 ml); the middle step was 30% Optiprep (6.9 ml), and the top step was 2.0 ml of TXNE. Gradients were centrifuged at 40,000 rpm for 20 h at 4°C in the SW50Ti rotor (Beckman). Seventeen fractions of 0.6 ml were harvested from the top to the bottom. To ensure quantitative recovery of proteins, the fractions were precipitated with 10% trichloroacetic acid in the presence of 200 μg of insulin per mL. The protein pellets were washed by vortexing with 1 ml of −20°C acetone to remove traces of trichloroacetic acid, before being dissolved in the SDS sample buffer.

### ChIP Assay

ChIP assay was performed according to the manufacturer's protocol using the Chromatin Immunoprecipitation Assay Kit (17,295, Millipore, Burlington, Mass, US). Briefly, 20 × 10^6^ C666-1 cells were used for each point. Cells were treated with 1 μg/ml PGN, LSD1, inhibitor (6 nM), or both together overnight. ChIP IgG was used as a negative control. The primers used are listed in Supplementary Table S1. No less than three biological replicas were acquired for the ChIP assay.

## Data Availability

The original contributions presented in the study are publicly available. This data can be found here: https://www.ncbi.nlm.nih.gov/geo/GSE194333).
