# Supplementary material for: Mechanisms of Anergic Inflammatory Response in Nasopharyngeal Carcinoma Cells Despite Ubiquitous Constitutive NF-κB Activation
Source: Front Cell Dev Biol. 2022 Jul 22;10:861916. doi: 10.3389/fcell.2022.861916 (PMC9353648; doi:10.3389/fcell.2022.861916)
Supplement: Supplementary file 1 [file Table1.DOCX]

**Supplementary information**

**Table S1. Primers used in this study.**

|  | **Genes** | **sequence(5’-3’)** |
| --- | --- | --- |
| Realtime RT-PCR | IL6 | F: GGTACATCCTCGACGGCATCT |
|  |  | R: GTGCCTCTTGCTGCTTTCAC |
|  | IL8 | F: ACTGAGAGTGATTGAGAGTGGAC |
|  |  | R: AACCCTCTGCACCCAGTTTTC |
|  | IL1α | F: GTTTAAGCCAATCCATCACTGATG |
|  |  | R: GACCTAGGCTTGATGATTTCTTCCT |
|  | CXCL2 | F:CGCCCAAACCGAAGTCATAG |
|  |  | R: AGACAAGCTTTCTGCCCATTCT |
|  | GAPDH | F:CTTCTGAGTTGCCCAGGAGACCACT |
|  |  | R:TCAACCACTCACACACACACAACCA |
| Realtime PCR | IL6 | F: TAGAGCTTCTCTTTCGTTCCCGGT |
|  |  | R: TGTGTCTTGCGATGCTAAAGGACG |
|  | IL8 | F: GGCCATCAGTTGCAAAT |
|  |  | R: TTCCTTCCGGTGGTTTCTTC |

**Table S2. Genes upregulated in NP69 upon treatment with PGN and streptococci, respectively, by KEGG pathway analysis.**

| NP69-PGN | NP69-streptococci |
| --- | --- |
| Cytokine cytokine receptor interaction | |
| Jak-Stat signaling | |
| Focal adhesion | |
| Adipocytokine signaling pathway | |
| Toll-like receptor signaling | |
| Tight junction | |
| ECM receptor interaction | |
| Leukocyte transendothelial migration | Neurotrophin signaling pathway |
| Cell adhesion molecules | T cell receptor signaling pathway |
| Intestinal immune network for IgA production | Apoptosis |
|  | TGF-beta signaling pathway |
|  | NOD-like receptor signaling pathway |
|  | Hematopoietic cell lineage |
|  | RIG-1 like receptor signaling pathway |
|  | Cytosolic DNA sensing pathway |
|  | Regulation of aotophagy |

**Table S3. Genes upregulated in NP69 upon treatment with LPS and PGN, respectively, by KEGG pathway analysis.**

| NP69-LPS | NP69-PGN |
| --- | --- |
| Neurotrophin signaling pathway | Cytokine cytokine receptor interaction |
| Phosphatidylinositol signaling system | Jak-Stat signaling |
| Apoptosis | Focal adhesion |
| Cell cycle | Leukocyte transendothelial migration |
| P53 Signaling pathway | Toll-like receptor signaling |
| Proximal tubule bicarbonate reclamation | Cell adhesion molecules |
|  | Intestinal immune network for IgA production |
|  | Adipocytokine signaling pathway |
|  | Tight junction |
|  | ECM receptor interaction |

**Table S4. Genes upregulated in NP69 and C666-1 upon treatment with streptococci by KEGG pathway analysis.**

| NP69-streptococci | C666-1-streptococci |
| --- | --- |
| Tight junction | |
| Cytokine cytokine receptor interaction | Leukocyte transendothelial migration |
| Jak-Stat signaling | Phosphatidylinositol signaling system |
| Focal adhesion | Wnt signaling pathway |
| Neurotrophin signaling pathway | Cell adhesion molecules |
| Toll-like receptor signaling | Hedgehog signaling pathway |
| T cell receptor signaling pathway |  |
| Tight junction |  |
| Apoptosis |  |
| Adipocytokine signaling pathway |  |
| TGF-beta signaling pathway |  |
| RIG-1 like receptor signaling pathway |  |
| NOD-like receptor signaling pathway |  |
| Hematopoietic cell lineage |  |
| ECM receptor interaction |  |
| Cytosolic DNA sensing pathway |  |
| Regulation of autophagy |  |

**Figure S1**

**
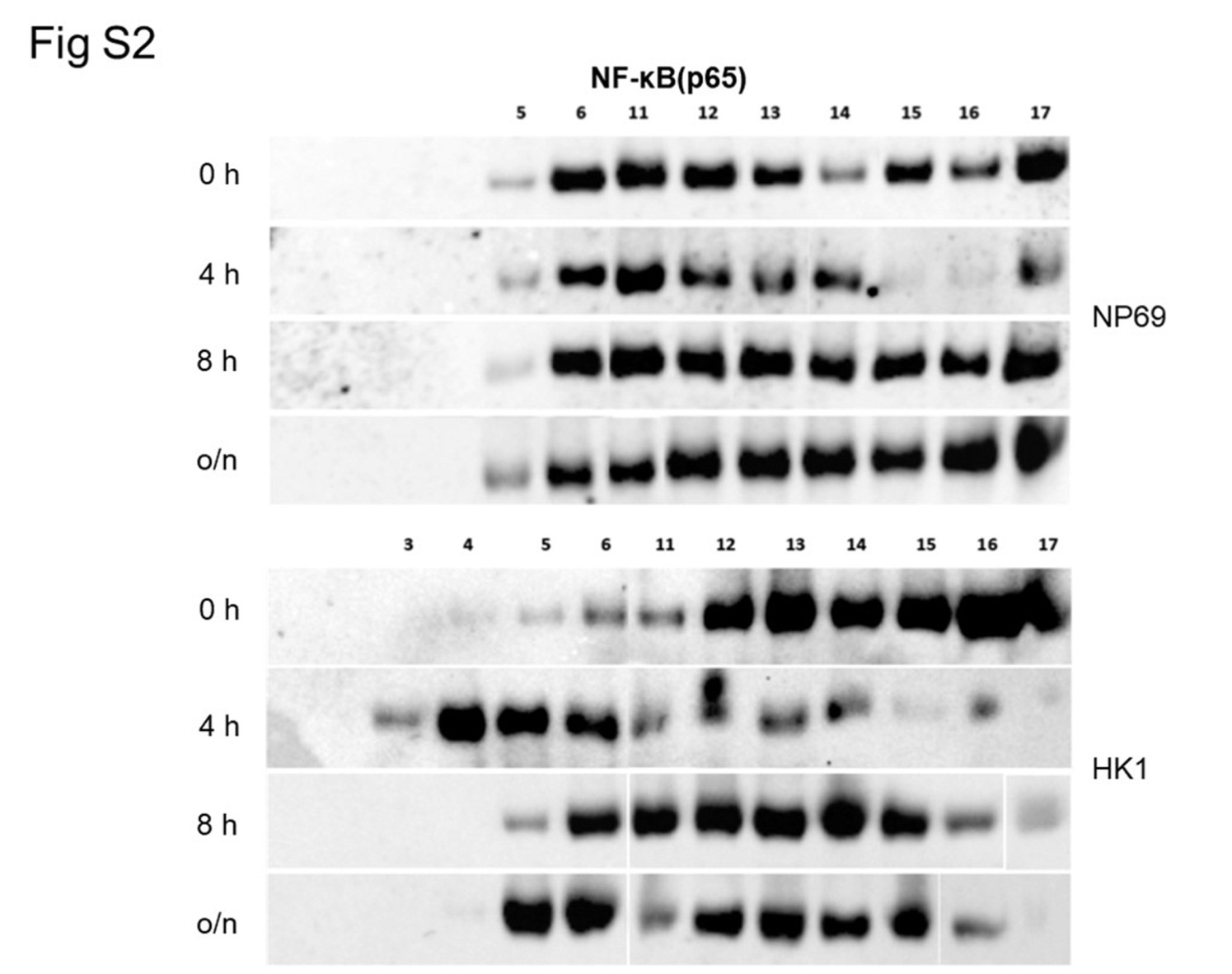
**

**Figure legend S1. NF-κB(p65) is associated with lipid droplets (LDs) in NPC cells.** Non-cancerous nasopharyngeal epithelial cell line NP69 and NPC cell line HK1 cells were treated with PGN for 2 hours, the media was changed, and the cells were harvested at 4 time points, including 0 hour, 4 hours, 8 hours and overnight. Cells were fractionated on a discontinuous density gradient to separate lipid-containing fractions. Fractions were analyzed by western blot for expression of NF-κB (p65).

**Figure S2**


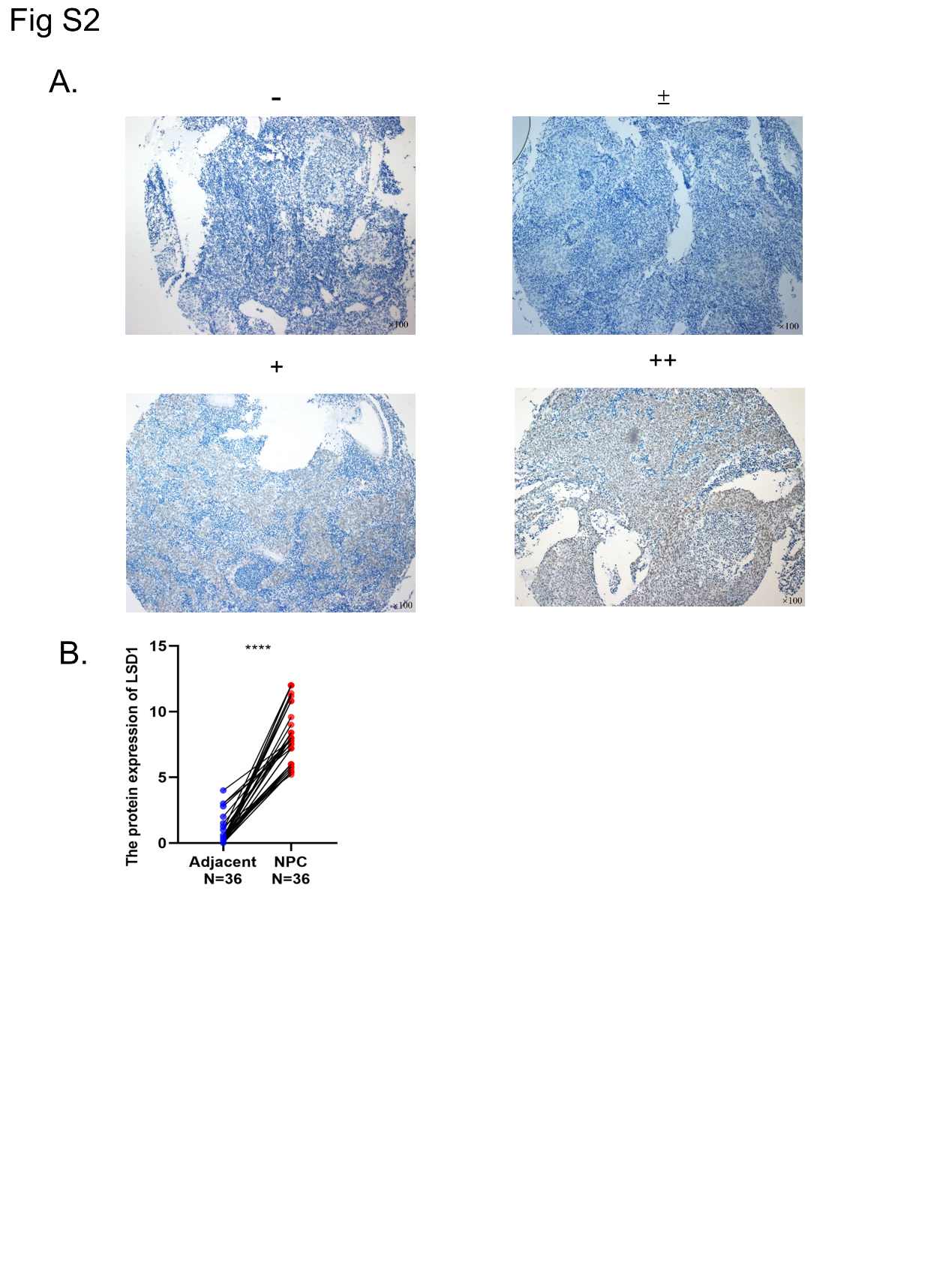


**Figure legend S2. LSD1 is overexpressed in NPC tissues.** A: Immunohistochemistry staining of LSD1 in 131 cases of NPC primary tissues. Presentative data shows the negative (-), weak positive (±), positive (+), and strong positive (++) staining of LSD1 (magnification ×100). B. Among the 131 NPC tissues, 36 cases contained adjacent normal epitheliums. The expression of LSD1 is scored and analyzed.****p*<0.001
